# Ionic Liquids Hybridization for Carbon Dioxide Capture: A Review

**DOI:** 10.3390/molecules28207091

**Published:** 2023-10-14

**Authors:** Asyraf Hanim Ab Rahim, Normawati M. Yunus, Mohamad Azmi Bustam

**Affiliations:** 1 Centre for Research in Ionic Liquid (CORIL), Institute of Contaminant Management, Universiti Teknologi PETRONAS, Seri Iskandar 32610, Malaysia; asyrafhanim92@gmail.com (A.H.A.R.); azmibustam@utp.edu.my (M.A.B.); 2 Department of Fundamental and Applied Sciences, Universiti Teknologi PETRONAS, Seri Iskandar 32610, Malaysia; 3 Department of Chemical Engineering, Universiti Teknologi PETRONAS, Seri Iskandar 32610, Malaysia

**Keywords:** ionic liquids, ILs hybridization, hybrid material, carbon dioxide capture

## Abstract

CO_2_ absorption has been driven by the need for efficient and environmentally sustainable CO_2_ capture technologies. The development in the synthesis of ionic liquids (ILs) has attracted immense attention due to the possibility of obtaining compounds with designated properties. This allows ILs to be used in various applications including, but not limited to, biomass pretreatment, catalysis, additive in lubricants and dye-sensitive solar cell (DSSC). The utilization of ILs to capture carbon dioxide (CO_2_) is one of the most well-known processes in an effort to improve the quality of natural gas and to reduce the green gases emission. One of the key advantages of ILs relies on their low vapor pressure and high thermal stability properties. Unlike any other traditional solvents, ILs exhibit high solubility and selectivity towards CO_2_. Frequently studied ILs for CO_2_ absorption include imidazolium-based ILs such as [HMIM][Tf_2_N] and [BMIM][OAc], as well as ILs containing amine groups such as [Cho][Gly] and [C_1_ImPA][Gly]. Though ILs are being considered as alternative solvents for CO_2_ capture, their full potential is limited by their main drawback, namely, high viscosity. Therefore, the hybridization of ILs has been introduced as a means of optimizing the performance of ILs, given their promising potential in capturing CO_2_. The resulting hybrid materials are expected to exhibit various ranges of chemical and physical characteristics. This review presents the works on the hybridization of ILs with numerous materials including activated carbon (AC), cellulose, metal-organic framework (MOF) and commercial amines. The primary focus of this review is to present the latest innovative solutions aimed at tackling the challenges associated with IL viscosity and to explore the influences of ILs hybridization toward CO_2_ capture. In addition, the development and performance of ILs for CO_2_ capture were explored and discussed. Lastly, the challenges in ILs hybridization were also being addressed.

## 1. Introduction

Natural gas and biogas offer a clean burning and play a significant role in various sectors such as electric power plants, household heating, transportation and industrial processes. However, due to the presence of acidic gases such as carbon dioxide (CO_2_) and hydrogen sulfide (H_2_S), the process of gas sweetening has become one of the most vital step in gas processing technology [1]. Generally, raw natural gas contains 20 mol% of acidic CO_2_. The presence of CO_2_ reduces the natural gas burning velocity due to its limited heating value [2]. Furthermore, the presence of moisture and CO_2_ leads to the formation of carbamic acid, which contributes to pipeline corrosion. Therefore, efficient CO_2_ capture technologies from natural gas are vital for enhancing the marketability of the end product and mitigating the emission of greenhouse gases. Generally, CO_2_ capture in natural gas is associated with extraction, processing and combustion. Technologies developed for capturing CO_2_ in natural gas including pre-combustion, post-combustion and oxyfuel. In the meantime, several CO_2_ capture techniques have been developed based on chemical absorption, physical separation, membrane separation and cryogenic distillation [3,4].

The usage of alkanoamines such as MDEA, DEA and MEA are by far the most developed process in gas sweetening. Amines offer a reliable and effective solution for CO_2_ capture, particularly in post-combustion capture applications due to their affordability, high CO_2_ absorption efficiency and selectivity [5]. Nevertheless, amines have low thermal stability and high volatility which renders them unsuitable for operation under high-temperature conditions. Consequently, the loss of amines during the absorption process becomes inevitable. Furthermore, the degradation of amines through oxidation or thermal processes resulted in the formation of chemical compounds that exhibits low absorption capacity such as organic acids, amides, dimers and trimers [6]. Besides that, aqueous amines consume high energy during the regeneration process due to the strong interaction between CO_2_ and the sorbent [7,8]. For instance, in the case of MEA regeneration, the physical treatment was conducted at 160 °C to desorb CO_2_. The process resulted in a substantial 95% loss of amines [9].

**Figure 1:**
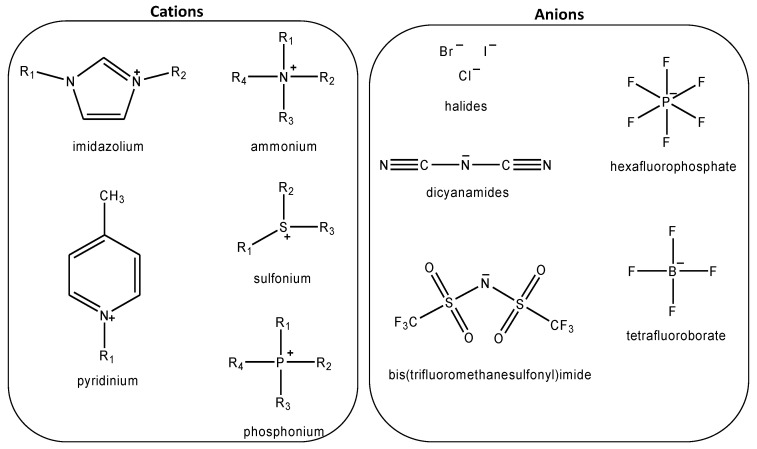
Examples of IL cation and anion.

Meanwhile, ionic liquids (ILs) are a class of molten salt that is formed by the combination of organic cation with organic or inorganic anion. They were initially introduced in 1914. However, the interest towards ILs started around 1997 after being identified as promising solvents for green technology. Since then, ILs have been applied in various applications including, but not limited to, wastewater treatment, drug design, biodiesel production, biomass pretreatment and as additives in lubricants [10,11,12,13,14]. In the meantime, ILs have emerged as a promising alternative sorbent for CO_2_ capture in the effort to find innovative solutions that are able to address the challenges associated with traditional amine-based capture methods. The development of ILs as sorbents in the field of CO_2_ capture is mainly attributed to their low vapor pressure values [15]. The utilization of non-volatile material such as ILs has garnered significant attention in addressing the solvent loss issue during absorption and regeneration process. Moreover, ILs are also known as designated solvents due to the wide range of possible combinations of anions and cations as depicted in Figure 1. This allows the properties of ILs to be tailored, thereby providing an opportunity to enhance the efficiency and sustainability of CO_2_ capture. Apart from that, ILs also possess several other unique properties including good thermal and chemical stability, broader liquidous range and wide electrochemical window [16,17]. To date, a wide range of ILs from different classes, such as Room-temperature ILs (RTILs), Task-specific ILs (TSILs), Dual-functionalized ILs (DFILs) and amino-acid ILs (AAILs), have been synthesized and investigated for CO_2_ capture. These ILs encompass various cations, including imidazolium, pyridinium, phosphonium, cholinium and ammonium [18,19,20,21].

## 2. ILs Utilization for CO_2_ Capture

The application of ILs for CO_2_ capture was initially reported by Blanchard and co-workers involving the synthesis of conventional RTILs, [BMIM][PF_6_] for high-pressure CO_2_ absorption [18]. [BMIM][PF_6_] displayed a promising outcome as it recorded 0.6 mol fraction of CO_2_ at 80 bar. The same research group also performed a study on phase behavior of ILs/CO_2_ system at high pressure in which the result showed that [BMIM][PF_6_] achieved 0.72 mol fraction of absorption capacity at 40 °C, and 93 bar [22]. Since then, numerous ILs have been screening while simultaneously studying the effect of anion and length of alkyl chain towards CO_2_ capture. Cadena et al. presented experimental and simulation studies for CO_2_ absorption using imidazolium-based ILs coupled with several anions, namely, hexafluorophosphate [PF_6_]^−^, tetrafluoroborate [BF_4_]^−^ and bistrifluoromethyl(sulfonyl)imide [Tf_2_N]^−^ [23]. The result demonstrated ILs with [Tf_2_N]^−^ displayed the best affinity towards CO_2_ compared to other anions. The same group also studied the effect of methyl group on C2 of imidazole cation of [EMMIM][Tf_2_N]. It has been found that the CO_2_ absorption capacity of [EMMIM][Tf_2_N] and [EMIM][Tf_2_N] were approximately 0.26 and 0.28 mol fractions, respectively, thus suggesting a minor influence of the cation on the absorption process. While numerous publications confirm the significant impact of anions on CO_2_ absorption, whether through experimental or simulation data [24,25], the study on the effect of ILs alkyl chain demonstrated that the CO_2_ absorption increased as the alkyl chains becomes longer [26,27].

As the field progressed, Task-Specific ILs (TSILs) were designed to tailor the properties of absorbent exclusively for enhancing CO_2_ capture performance. Normally, TSILs comprise of amine moiety, which is expected to assist in improving CO_2_ absorption through chemisorption mechanism. A study by Sharma et al. demonstrated the CO_2_ absorption capacity by [2-AEmim][Tf_2_N] reached 0.49 mol CO_2_/mol IL at 1.6 bar under 30 °C [28]. The research revealed that the CO_2_ absorption process in amine-functionalized ILs follows a chemisorption mechanism. The analysis of the functional groups of [2-AEmim][Tf_2_N]-CO_2_ indicated the presence of new peaks at 1666 cm^−1^, which can be attributed to the carbamate (=C=O) group. This finding was supported by ^13^C NMR spectroscopy, where new peaks at 162.3 and 56.5 ppm were observed, corresponding to the carbamate carbon and the methylene group of the carbamate nitrogen atom, respectively. Besides that, the work revealed ILs with [Tf_2_N]^−^ recorded higher CO_2_ absorption capacity than [BF_4_]^−^ and dicyanamide [DCN]^−^. Additionally, Hussain synthesized pyridinium-based ILs consisting of ether-functionalized groups combined with cyano-based anions, thiocyanate [SCN]^−^, [DCN]^−^ and tricyanamide [TCN]^−^ [29]. This result demonstrated that the solubility of CO_2_ increased with an increasing number of cyanide groups. On the other hand, the anion provided a significant effect towards CO_2_ absorption though with the presence of functionalized groups.

Apart from that, ILs functionalization is not only limited to the cation site but also encompasses the anion as well. Up to the present, numerous studies have been conducted to explore the potential of ILs containing carboxylate, azolate, phenolate and alkoxide anions for CO_2_ absorption [30]. A study by Keller et al. stated one of their azolate anion-functionalized ILs, [P_2228_][6BrInda], which was also known as aprotic heterocyclic anion (AHA), demonstrated nearly equimolar CO_2_-ILs uptake at 0.833 bar, 332 K [31]. This value is higher than what was previously reported for the conventional [HMIM][Tf_2_N] and [BMIM][OAc]. It was found that the CO_2_ absorption was affected by the electron density of reactive nitrogen. Keller and co-workers showed that the CO_2_ absorption increased from 0.91 for [P_2228_][4BrInda] to 0.98 molCO_2_/mol ILs for [P_2228_][6BrInda], and the reason was attributed to the fact that [6BrInda]^−^ owned more electron withdrawing group (EDW) than that of [4BrInda]^−^. Consequently, the electron density increased with the presence of EDW, thereby enhancing the CO_2_ absorption capacity of the ILs. In the meantime, the design of ILs for highly efficient CO_2_ chemisorption was conducted by introducing TSILs comprise of commercial phenol as the starting material. Zhao reported a series of low viscous diamino protic ILs couples with phenolic anion prepared via direct neutralization for CO_2_ absorption. The CO_2_ solubility significantly increased with rising pressure in the low-pressure region, indicating that CO_2_ absorption occurred through both chemical and physical mechanisms [32]. Furthermore, their [DMAPAH][4F-PhO] exhibited a CO_2_ absorption capacity of up to 3.99 mol CO_2_/kg ILs. The recyclability test that was conducted up to five times showed a slight decline in CO_2_ absorption, indicating the promising potential of ILs.

The research in CO_2_ was broadened to the development of polymeric-ILs with the goal of enhancing the stability, durability and capability to control the macromolecular framework of sorbents. Apart from that, the copolymerization of polymeric ILs with other polymers allows for the attainment of a high surface area, which is favorable for gas capture applications. In one of the earliest examples, Tang and colleagues synthesized polymeric-ILs using imidazolium-based IL as monomers [33]. A comparative study revealed that [PVBIM][BF_4_] exhibited a CO_2_ absorption capacity of 0.305 wt%, while [BMIM][BF_4_] had a capacity of 0.256 wt%. Additionally, they observed that all the polymeric ILs, namely, [PVBIM][PF_6_], [PVBIM][BF_4_] and [PBIMT][BF_4_], demonstrated rapid CO_2_ sorption compared to the conventional [BMIM][BF_4_]. While [BMIM][BF_4_] required 400 min to reach its full capacity, all other polymeric-ILs achieved maximum absorption within just 30 min. Generally, the formation of a porous structure within the polymer matrix could facilitate the diffusion of CO_2_ molecules into the polymeric-ILs, which in turn leads to rapid absorption kinetics and efficient CO_2_ capture. Subsequently, numerous polymeric ILs with various cations, including pyridinium, ammonium and phosphonium, have emerged as promising solutions for the improvement in CO_2_ uptake capacity. Another study involves the synthesis of pyridinium-based polymeric-ILs in which the polymer ILs [MDAP][TF_2_N]-PDC exhibited 1.39 wt% of CO_2_ absorption at 1.0 bar under temperature 25 °C [34]. On the other hand, the recent trend in the field of polymeric ILs involves the development of hyper-cross-linked poly (ILs), (HCL-PILs). Sang and Huang synthesized HCL-PILs by introducing benzimidazole as additional monomer via Friedel-Crafts alkylation [35]. The analysis conducted using BET indicated that HPIL-Cl (1) exhibited a CO_2_ uptake of 5.3 wt%, which was higher than the 4.5 wt% observed for HCP-Cl. According to the authors, the enhancing in CO_2_ capture in HPIL-Cl (1) was due to the presence of imidazoline and imidazolinium cations that exhibit strong electrostatic force as the main configurations for the polymers.

AAILs have emerged as a distinct class of ILs as they offer a promising platform for the development of biodegradable absorbent for CO_2_ capture. Furthermore, the presence of amine and carboxylic functional groups in amino acid may facilitate strong interactions and selective binding with CO_2_ molecules. The utilization of AAILs was firstly introduced in a study by Zhang and co-workers in 2005, in which they studied the application of phosphonium-based ILs coupled with several amino acids as anion supported porous silica for CO_2_ capture [36]. This also serves as one of the first examples of IL hybridization process, even though the term “hybridization” may not have been widely recognized initially. The same group also performed the CO_2_ absorption by using [P(C_4_)_4_][Gly] that contained 1% mass fraction of water and the CO_2_ uptake by the IL amounted to ~0.12 mole fraction. However, it was not until 2015 and onwards that AAILs gained significant attention from researchers, as evidenced by the surge in publications related to AAILs for CO_2_ capture and its mechanism [19,37,38,39]. A study by Yang et al. showed the CO_2_ capture capacity of their AAILs were more than 0.5 mol CO_2_/mol IL [40]. It has also been observed that CO_2_ absorption mechanism by AAILs was affected by the types of amino acid. For example, more than 95% of absorbed CO_2_ from a total of CO_2_ absorption capacity by [P_66614_][Pro] and [P_4444_][Pro] were achieved via 1:1 reaction mechanism in which 1 CO_2_ molecule react with 1 amine molecule. Apart from [Pro] ILs, other ILs exhibited a contribution of over 20% to the CO_2_ absorption capacity through the reaction involving 1 CO_2_ molecules per 2 amines molecules (1:2 mechanism). Various works also demonstrated the amount of amine sites presence in ILs chemical structure was found to have an impact on the capacity of CO_2_ absorption [19,38,41,42]. For example, among the pyridinium-based ILs with different amino acid anions, such as arginate [Arg]^−^, lysinate [Lys]^−^, alanate [Ala]^−^, and thryrosinate [Thy]^−^, the [B_4_MPyr][Arg] demonstrated remarkable CO_2_ absorption capacity [38]. This can be attributed to the presence of two primary and two secondary amines in [Arg]^−^ structure, thus providing more active sites to be interact with CO_2_ molecules [38].

In the meantime, dual-functionalized ILs (DFILs) refers to the ILs class with two similar or different functional groups present in both of their cation and anion. Examples of dual-functionalized ILs are [C_1_ImPA][Gly], [TEPA][Im] and [TETAH][Pz] [43,44,45]. The main purpose in the synthesis of dual-functionalized ILs is to provide an additional active site for the reaction to occur. As stated by Zhang et al., the earliest type of dual-functionalized ILs used for CO_2_ capture primarily comprises of a cation with amino-functionalization and an amino acid anion. Lv and co-workers prepared an aqueous solution containing [C_1_ImPA][Gly] in which the CO_2_ capacity recorded at 30 °C was 1.23 mol CO_2_/mol ILs [43]. Despite its excellent performance, it was reported that the absorption capacity of pure [C_1_ImPA][Gly] was higher than that of its aqueous solution. The presence of water in aqueous [C_1_ImPA][Gly] may negatively impact the CO_2_ absorption due to the competition between water and CO_2_ molecules for active sites in the ILs. However, pure dual-amino functionalized ILs are reported to be highly viscous, thus discouraging their neat use in CO_2_ capture. Therefore, the incorporation of other functionalized groups such as ether is proposed to reduce the ILs viscosity. The rotational flexibility that is caused by the presence of oxygen atom is expected to reduce the Ils viscosity. For instance, ether-functionalized choline tethered amino acid, [N_1,1,6,2O4_][Lys] was synthesized and has a viscosity value of 184 mpa-s at 80 °C, while [N_6,6,6,14_][Lys] was reported to have a viscosity value of >200 mpa-s at the same temperature. CO_2_ absorption measurement by using low viscosity [N_1,1,6,2O4_][Lys] was found to reach equilibrium conditions within 3.5 h with the absorption value of 1.62 mol of CO_2_/mol ILs [46]. In comparison, ether-free ILs such as [N_6,6,6,14_][Lys] and P_6,6,6,14_][Lys] recorded significantly longer equilibration time, between 24 and 48 h, during CO_2_ absorption.

One of the purposes of designing AAILs is to overcome the high viscosity problem of ILs. However, the viscosity of AAILs have drastically increased after CO_2_ absorption due to formation of hydrogen bond [47]. This finding has prompted researchers to develop a new class of ILs known as protic ILs (PILs). PILs are synthesized through proton transfer between a Bronsted acid and base. In addition to their low viscosity, PILs require a simple synthesis procedure without complicated purification steps. One of early examples of PILs for CO_2_ is demonstrated by Wang and co-workers in 2010 [48]. The study demonstrated that their superbase PILs [MTBDH^+^]_2_ [HFPD^2−^] exhibited a remarkable CO_2_ absorption capacity of 2.04 mol CO_2_/mol ILs, despite being in a gel form. This can be attributed to the presence of two CO_2_-reactive sites in the PIL. On the other hand, Li et al. prepared three low-viscous PILs for CO_2_ absorption [49]. It was determined that [TMGH][Pyrr] with the largest basicity recorded the highest CO_2_ absorption capacity of 0.66 mol CO_2_/mol IL and took 60 min for [TMGH][Pyrr] to reach equilibrium condition. However, [TMGH][Im] that possessed the second highest basicity exhibited rapid absorption rate as its reaching equilibrium within 20 min with CO_2_ absorption capacity of 0.64 mol CO_2_/mol IL.

Based on this section, it can be summarized that numerous efforts have been made by focusing on optimizing IL properties to achieve higher CO_2_ absorption capacities, improved selectivity and enhanced stability. Researchers have explored various strategies such as modifying ILs structure by incorporating numerous functional groups into the anion or cation with the main goal of improving the CO_2_ capture performance. Meanwhile, regardless of the class of ILs, the anion has been observed to play a significant role in CO_2_ absorption. Table 1 lists several ILs according to ILs classes studied for CO_2_ capture. In general, ILs could become a new alternative in CO_2_ capture technology. In addition to ILs low volatility and thermal stability, the ability to customize their properties for specific applications has emerged as a significant factor that attracts researchers’ attention to further explore their potential, particularly in the field of CO_2_ capture.

### Challenges in ILs Viscosity towards CO_2_ Capture

As reported by Hospital-Benito, an increase in solvent viscosity has resulted in a 30% reduction in CO_2_ recovery [51]. Thus, viscosity remains a critical factor influencing the effectiveness of ILs in capturing CO_2_. Though ILs offer promising solutions in terms of low volatility and recyclability, their viscosity issue poses several challenges when considering realistic operating conditions within industrial scales. In general, highly viscous ILs will lead to slow diffusion of CO_2_ into the sorbent which, in turn, will cause reduction in mass transfer and, consequently, will increase energy consumption. Additionally, regenerating ILs necessitates the provision of heat to facilitate the desorption process. In the case of highly viscous ILs which have low heat transfer, additional energy input may be required thereby impacting process efficiency. Meanwhile, a study by Morta-Martinez revealed that viscosity and heat capacity have a direct effect on the capital and operating cost as those properties involve in both the energy requirement and the design of absorption column [15]. For instance, a heat generator used to increase the operating temperature is employed to reduce the viscosity of ILs, thereby enhancing the rate of CO_2_ diffusivity [52,53]. Nevertheless, it is worth noting that this approach can have a counterproductive effect as the solubility selectivity of CO_2_ in ILs typically decreases with increasing temperature [54]. In summary, the viscosity of ILs plays a central role in experimental and process design for CO_2_ absorption. Therefore, the aim of this paper is to discuss the current development in capturing CO_2_, particularly in the efforts to overcome the viscosity challenge.

## 3. ILs Hybridization Strategy

The preceding section summarizes the evolution in ILs development for CO_2_ capture. It is undeniable that ILs have shown excellent potential to be alternative sorbents for CO_2_ removal. However, the utilization of ILs in real application has been hampered due to several challenges. ILs demonstrate high viscosity property which will impede the mass transfer by inhibiting gas diffusion into liquid, thus reducing the efficiency in capturing CO_2_ [55]. Some functionalized ILs including AAILs, sulfone and acetate were reported to display a significant increase in viscosity after CO_2_ absorption [56]. This effect will be more pronounced at industrial level where the need of rapid CO_2_ removal is required. Besides that, several ILs utilize specific precursors which involve extensive purification during their synthesis procedure, and this is seen to be a major drawback [57]. For example, ILs with aprotic heterocyclic anion (AHA) have been recognized as a promising candidate for CO_2_ capture due to their capability to react stoichiometrically and reversibly with CO_2_ while avoiding the increase in viscosity as being observed in AAILs [58]. Nevertheless, the synthesis of AHA-ILs requires multiple steps including anion exchange and neutralization reaction to yield the desired ILs. In addition to the time-consuming anion exchange process, effective CO_2_ capture necessitates ILs to undergo thorough drying process, which typically takes about a week. In turn, these present economic challenges for industrial application due to high production cost. Other than that, the complexity in IL preparation is not only limited to the selection of cation and anion combination, but also involves the intricate nature of ILs synthesis as parameters that prove to be effective on a small scale may not directly applicable to larger-scale production. Factors such as mixing speed, reaction temperature and synthesis period need to be carefully considered due to their significant impact on the process.

Therefore, to address the limitation of ILs in CO_2_ capture, ongoing research focuses on exploring new materials. One of the popular alternatives is to produce a hybrid material consists of IL with the goal to enhance properties or functionalities beyond what either component can achieve individually. According to Luo and co-workers, “hybridization” is a biological term referring to artificial integrations of two distinction species [59]. In the context of ILs, the term “ILs hybridization” refers to the process of incorporating substances such as mesoporous silica, metal organic frameworks (MOFs), activated carbon (AC), carbon nanotube (CNT) and deep eutectic solvent (DES) with ILs to produce new materials with superior property [60,61,62]. For instance, Roh et al. employed this term to describe the combination of their IL with carbon nanotube (CNT), while Huang and co-workers used it in their work related to ILs encapsulation with shell of polyurea and alkylated graphene oxide [63,64]. Hybridization of ILs allows the material to have more versatility and adaptability for specific application. ILs hybridization also minimizes the amount of ILs required, thereby effectively reducing the associated cost [59]. Furthermore, the hybridization of ILs with other materials, such as amine blending, has proven to be an effective approach in addressing the issue of viscosity [65].

### 3.1. Methods in ILs Hybridization

There are several methods that can be applied to hybridize IL with other materials. In pursuit of enhancing the intended application, the method selection may vary based on several factors such as the desired properties of the hybrid materials and the compatibility of the materials involved.

#### 3.1.1. Blending/Mixing

Blending or mixing is one of the common techniques in the ILs hybridization process. Normally, blending is carried out by mixing ILs with one or more liquid material at room temperature. To date, there are numerous types of IL binary mixtures or hybrid solvents that have been prepared comprising of IL-water, IL-DES, IL-organic solvent and IL-amine [66]. For example, Zainul Anuar and co-workers prepared IL-hybrid solvent by adding 2.0–20.0 wt% of their [TBP][MeSO_3_] into aqueous solution of MEA [67]. Another example is the preparation of biphasic mixture consisting of [TETAH][Ly], water and ethanol [68].

#### 3.1.2. Immobilization

The immobilization of ILs into porous solid has been frequently conducted in catalytic application with the aim to transfer the desired properties of liquid into solid catalyst. Nowadays, the immobilization process has been extended into various applications such as gas adsorption, wastewater treatment and drug design. In general, immobilized ILs contain three main parts which are porous support, functional or active group and catalyst or co-catalyst. As stated by Li et al., ILs can be immobilized through three different synthetic pathways, via active anion, via cation groups and via solid support [69]. The preparation of immobilized ILs via active anion groups is conducted through incipient wetness or immersed method. It involves the incorporation of reactive anion groups within a solid support matrix. During the incipient wetness method, the porous material is immersed in pre-prepared functionalized ILs containing active groups. Through this process, ILs are confined within the pores of the porous material by a covalent bond resulting from the interaction between the active anion and functional group present on the solid support. On the other hand, the synthesis of immobilized ILs via a cation group is performed through grafting of covalent anchoring method. In this method, the organic anchor group that is covalently attached to the surface of solid support acts as a cation, thus allowing the formation of ILs thin film on the support surface. Another approach involves encapsulation of ILs within the protective shell of solid support [70]. The encapsulation of ILs can be achieved using various techniques, namely, microencapsulation, nanoencapsulation and polymer-based encapsulation. 

#### 3.1.3. Wet Impregnation

The wet impregnation method is widely regarded as one of the simplest approaches for preparing ILs hybrid materials primarily due to the flexibility of material combinations it offers. In wet impregnation, ILs are introduced into the porous of solid material using solvents such as water or organic solvents. The reaction is conducted at room temperature followed by gradual temperature elevation to facilitate solvent evaporation. 

#### 3.1.4. Polymerization Technique

Another method to produce IL-hybrid material is by polymerization technique. This material, which can also be referred to porous ILs, is synthesized through in situ polymerization, wherein ILs can act as monomers or undergo copolymerization with other monomers in the presence of a solid template. For example, Ding and Jiang prepared their hybrid material by simultaneously introduce [VEIMBr] and cross linker of *ortho*-divinylbenzene (*o*-DVB) into MOF which subsequently be copolymerized to produce poly-ILs-hybrid porous material [71]. 

#### 3.1.5. Ionothermal Synthesis

Ionothermal synthesis is a method for synthesizing hybrid material using ILs simultaneously as the reaction medium and directing agent. Unlike traditional methods which utilize organic solvents, the selection of ILs as reaction medium affected the architecture of final product. Vaid and co-workers reported the synthesis of cobalt benzenetricarboxylate MOF by using two different solvents, [EMIMBr], [EMIM][Tf_2_N] and equimolar mixture of [EMIMBr]:[EMIMTf_2_N], resulting in three MOFs with different structural template [72]. In the meantime, Table 2 summarizes the advantages and disadvantages of ILs hybridization technique. 

## 4. Application of ILs Hybrid Material in Carbon Dioxide

The idea on ILs hybridization was first proposed as researchers began to explore the incorporation of this designated solvent into solid matrixes. Since then, numerous studies focusing on ILs’ incorporation into solid porous materials such as zeolites, silica and AC for CO_2_ adsorption have been published [81,82,83]. Subsequently, the research has been extended to blended solvents by combining ILs with different liquid materials including deep eutectic solvents (DES) and commercial amines [66,84]. In recent years, there has been notable shift on ILs hybridization leading to the development of IL-membrane for selective separation [85]. Therefore, this section will explore various ILs hybridization strategies that are utilized to enhance CO_2_ capture.

### 4.1. ILs/Amines

The utilization of amine for CO_2_ capture via chemical absorption process dates to 1930. Over the years, various amines have been extensively used in industrial application due to high absorption capacity caused by strong chemical reactivity with CO_2_. Examples of common amines used are DEA and MEA. These two primary amines exhibit strong interaction with CO_2_. However, due to strong chemical interaction of amines with CO_2_, the desorption process requires significant energy input which can impact the operational cost of the whole system. Therefore, current research for amine usage in CO_2_ focuses on developing hybrid absorbent with minimum energy consumption during regeneration process. Apart from that, the incorporation of amines is anticipated to overcome the viscosity issue that is primarily associated with ILs. Moreover, blending amines with ILs can enhance the selectivity of the solvent towards CO_2_, which may improve the efficiency of the capture process and reduce the energy required for subsequent separation and regeneration steps.

In another study, two imidazolium-based ILs, namely, [BMIM][BF_4_] and [BEIM][BF_4_], were blended with aqueous MEA and MDEA [52]. The addition of amines reduced the viscosity of absorbent and significantly enhanced the CO_2_ absorption capacity. For example, [BMIM][BF_4_]/MDEA resulted in a higher CO_2_ absorption capacity of 0.0526 g CO_2_/g absorbent, whereas the pure ILs achieved only 0.0168 g CO_2_/g absorbent. Additionally, [BEIM][BF_4_]/MDEA and [BEIM][BF_4_] demonstrated CO_2_ absorption capacities of 0.0517 and 0.0174g CO_2_/g absorbent, respectively. The absence of peak attributed to carbonyl of carbamate on ^13^C NMR spectrum of ILs/MDEA spectra analysis showed the CO_2_ capture by using blending mixtures occurred via physical absorption. The study on sorbent regeneration showed [BEIM][BF_4_]/MDEA utilized less energy compared to aqueous amine absorbent. Theoretically, the interaction between the absorbent and CO_2_ in physical absorption is primarily based on weak intermolecular forces, which can be readily weakened or disrupted under moderate process conditions. This resulted in low energy input for CO_2_ desorption and regeneration compared to chemically driven absorption processes.

Li et al. investigated CO_2_ absorption at high pressure by using PILs blended with EDA or DETA [86]. In their work, the mixture of amine/PILs was prepared by extensively mixing various mass ratio of PILs: amine (1:1, 0.5:1, 1:0.5) at room temperature for 24 h. It has been observed that the viscosity of all blended mixtures is lower than the viscosity of pure ILs. The CO_2_ absorption was initially performed at 30 °C and 1 bar, and the result indicates [DMAPAH][Oac]-EDA exhibited 0.260 g CO_2_/g absorbent, which is higher compared to the CO_2_ absorption capacity demonstrated by [DMAPAH][Oac] alone. The experimental results also show that the CO_2_ absorption increased with the ratio of EDA in [DMAPAH][Oac]. This indicates that EDA is an excellent CO_2_ absorbent; however, its performance is limited due to its low thermal stability. The thermal stability of the absorbent has been improved particularly when ILs have been introduced in the mixture ratio of [DMAPAH][Oac]:EDA (0.5:1). The study was further explored by performing CO_2_ absorption at temperature range 20–60 °C using [DMAPAH][Oac]-EDA under atmospheric pressure. The experimental data indicated an increase in CO_2_ absorption capacity with the value of 0.295 g CO_2_/g IL at 50 °C. Addition of 20 wt% water into the mixture of [DMAPAH][Oac]-EDA had resulted in the absorption of 0.2229 g CO_2_/g absorbent. According to the authors, the CO_2_ absorption by [DMAPAH][Oac]-EDA involves the transfer of zwitterion into carbamate, which, in the presence of water, was further hydrolyzed to form bicarbonate. The presence of water not only accelerates the transfer rate, but also increases the primary amine availability through hydrolysis of bicarbonate. This, in turn, enhances the CO_2_ absorption rate and capacity. Other than that, the recyclability study showed the CO_2_ absorption capacity by [DMAPAH][Oac]-EDA exceeded the value of 0.2 g CO_2_/g IL even after it had been recycled three times. However, a decrease in performance was observed, which could be attributed to insufficient desorption and oxidative degradation of EDA.

Furthermore, Zalewski et al. performed a study using a simplex centroid design to investigate the experimental behavior of ternary mixture containing ILs, amine and water towards CO_2_ absorption capacity [87]. Two ILs, namely, [BMIM][Oac] and [EMIM][OcSO_4_], were chosen to be combined with amines that possess different basicity properties. The statistical analysis indicated that the concentration of ILs in the ternary mixture had a minimal influence on CO_2_ solubility, in which each ILs exhibited a different effect. The addition of [EMIM][OcSO_4_] to the binary mixture of amine/water resulted in a decrease in CO_2_ absorption capacity, while the opposite effect was observed upon the introduction of [BMIM][Oac]. Furthermore, a notable effect can be observed upon addition of ILs into a highly basic amine such as DBU. Analysis of NMR data revealed the formation of a newly attached carboxylic group to the C2 carbon of the imidazolium ring in [EMIM][Oac], which indicates the existence of chemisorption mechanism. Meanwhile, CO_2_ was specifically reacting with the bicarbonate generated through the reaction between the amine and water in DBU: [EMIM][OcSO_4_]: water (1:1:1) mixture. An additional study on amine degradation was carried out by focusing on [EMIM][OcSO_4_]/amine mixtures. The alkylation of [OcSO_4_]^−^ anion was found to cause the rapid degradation of MEA and DEA. This degradation was observed to occur more rapidly in amines in the absence of CO_2_. However, the degradation of DBU and DABCO were not affected by the presence of CO_2_. Moreover, the recyclability study demonstrated that the mixture containing DBU maintained its absorption capacity with minimal loss over multiple cycles. This marks it as a highly promising absorbent system for CO_2_ capture.

### 4.2. ILs/Activated Carbon

The usage of activated carbon (AC) for CO_2_ capture had been widely applied due to its porous structure, highly resistant towards toxic environment and complete adsorption reversibility during regeneration process [88,89]. An abundant source of biomass available as AC feedstock such as coconut coir, palm shell, nut shell and bamboo simultaneously provide a major solution to our agricultural waste management. Numerous studies related to the application of these carbonaceous materials have been conducted, in which excellent adsorption capacity was observed. For example, Ogungbenro and co-workers utilized AC from date seed for CO_2_ adsorption and the CO_2_ loading capacity exhibited by the date seed AC is comparable to commercial AC. However, though AC provides larger surface area for CO_2_ capture, its inert surface chemistry led to low capacity at low partial pressure [90]. Additionally, the presence of water vapor in flue gas has caused AC to exhibit low CO_2_ adsorption selectivity [91]. Therefore, the modification of AC through immobilization of ILs via grafting or impregnation methods is introduced with the aim to improve the affinity of the carbonized adsorbent toward CO_2_. 

Etho et al. functionalized two commercial ACs with different textural properties, F600-900 and RGC30, with [HMIM][BF_4_] and [EMIM][Gly], respectively [92]. The impregnation process of AC with [HMIM][BF_4_] and [EMIM][Gly] was conducted by using bath stirred system in ethyl acetate and methanol, respectively. After a week, the impregnated solid was collected by filtration. All functionalized AC was dried overnight at 70 °C. Then, AC/[HMIM][BF_4_] was further dried by purging N_2_ into column packed with impregnated AC, while for AC/[EMIM][Gly], the adsorbent was placed under vacuum at 100 °C for 5 h. It has been observed that the CO_2_ adsorption capacity of F600-900/[HMIM][BF_4_] and RGC30/[HMIM][BF_4_] was lower than raw AC at low temperature. However, the difference in CO_2_ adsorption capacity between impregnated adsorbent and raw AC was relatively low at 80 °C due to the insignificant effect of pores clogging caused by high reaction temperature. On the other hand, while the presence of [EMIM][Gly] did not induce the adsorption capacity of CO_2_ for F600-900 and RGC30 at 30 °C, an improvement was begun to be seen at 50 °C. According to authors, the presence of an amino group in [EMIM][Gly] may form carbamate with CO_2_, a mechanism that is similar to amine-based solvent reaction thus resulting in a comparable performance of functionalized samples with raw AC. Consequently, this contributed to the large CO_2_ adsorption capacity of F600-900/[EMIM][Gly] and RGC30/[EMIM][Gly] at 80 °C. Thus, impregnating AC with amino acid base IL shows a promising result for CO_2_ capture. The modification of IL structure by amine functionalization through amino-based IL with intention to mimic the commercial amine usage but with better thermal stability and heat capacity values is expected to benefit this application. On the other hand, incorporating AC with [HMIM][BF_4_] is found to be unsuitable for CO_2_ removal from flue gas as the presence of IL was unable to counteract the reduction of AC micropore.

Meanwhile, He and co-workers studied the effect of different methods, namely, impregnation and grafting, for AC surface functionalization [91]. In this work, the immobilized AC was prepared with different loading of [P_8883_][Tf_2_N]. In the impregnation method, AC was added into mixture containing IL and anhydrous ethanol and the mixture was stirred at room temperature for 24 h. The solid was filtered and dried under vacuum at 80 °C for 24 h. For the grafting technique, the mixture containing anhydrous ethanol, AC and ILs was stirred at reflux temperature for 36 h followed by extraction using acetone via Soxhlet extraction. The grafted AC was dried under vacuum at 95 °C for 8 h. Based on morphology analysis, the pore structure of AC/IL prepared by grafting method is more porous than AC/IL prepared using impregnation technique. High loading of IL into AC during impregnation process led to pore blockage. Besides that, the result indicated that the modified AC exhibits better CO_2_/N_2_ selectivity than raw AC. Data analysis had revealed impregnated AC showing high CO_2_/N_2_ selectivity due to higher IL loading compared to AC prepared via grafting method. Further observation on breakthrough curve showing the CO_2_ absorption capacity increased as pressure increased. Other than that, grafted AC/ILs showed higher CO_2_ absorption capacity than raw AC and impregnated AC due to less pore blockage by ILs compared to the other two samples. 

In another study, Shahrom and co-workers conducted a study on the effect of amine-functionalized using protic and amino ILs for AC impregnation towards CO_2_ adsorption [93]. The impregnated AC was prepared using physical immobilization with different percentage of ILs loading. Initially, an appropriate amount of AC was added into the mixture of IL and methanol. The mixture was gently shaken at room temperature for 24 h and methanol was slowly removed under vacuum condition to ensure successful immobilization. The resulting impregnated AC was then dried at 110 °C for 2 days. The surface area and pore volume of impregnated AC had reduced, thus indicating ILs’ successful impregnation. On the contrary, the AC/ILs had more CO_2_ adsorption capacity than raw AC due to the increment in pore size resulting from addition of smaller micropore filling by ILs. Besides that, due to acidic nature of CO_2_, it was expected that the presence of two amine functionalities at ILs with [Arg]^−^ and [Lys]^−^ anion on AC will provide an extra active site for gas adsorption compared to glycinate [Gly]^−^. However, the CO_2_ adsorption by AC/[VBTMA][Arg] and AC/[VBTMA][Lys] is lower than AC/[VBTMA][Gly]. Low CO_2_ adsorption capacity in [Arg]^−^-and [Lys]^−^-based ILs is possibly due to the blockage of internal pore structure caused by the presence of extra amines group. This, in turn, prevented the CO_2_ access into ILs active site. 

### 4.3. ILs/Mesoporous Silica

Due to its exceptional features, namely, highly ordered pore structure, large surface area and uniform pore size, mesoporous silica had become a center of attention in research field since late 1970s. Generally, mesoporous silica such as SBA-16, MCM-48 and MCM-41 are greatly favored for applications such as gas separation due to high BET surface area and pore sizes. Furthermore, the presence of silanol groups that act as adsorption main sites [94] combined with their capability to functionalize with various organic molecules [95] has assisted in elevating the performance of mesoporous silica in CO_2_ capture. The existence of a hydroxyl group also allows amine functionalization, thus enhancing the CO_2_ affinity in silica. Several studies related to the functionalization of various mesoporous silica with amine have been conducted with the aim to improve the material ability in CO_2_ adsorption. Ullah and co-workers introduced amine functional groups onto pores of SBA-15 [96]. The modified SBA-15 adsorbed 1.6164 mmol/g of CO_2_ at 1 bar, which is twice as much as the amount adsorbed by pristine SBA-15 due to high affinity effect of CO_2_ towards amine functional groups. Despite the promising result, there are still significant limitations which could contribute to the reduction in CO_2_ capture capacity by amine modified silica. For example, an ambient steam exposure led to degradation of the modified mesoporous silica structure due to amine evaporation, thus reducing the CO_2_ adsorption performance [95]. Due to the low vapor pressure of ILs, their confinement within the mesoporous silica structure is expected to improve the stability and regenerability of solid adsorbent.

Polesso and co-workers studied the effect of ILs immobilization with different anions on commercial SBA-15 9S (SBA) and silica obtained from rice husk (SIL) towards CO_2_ separation process [97]. Apart from becoming a good alternative to solve ILs viscosity issue, the loss of grafted or immobilized solvent can be avoided due to the low vapor property of ILs. In this work, each silica was immobilized using [BMIM][Cl] and [BMIM][OAc] through the wet impregnation method. The immobilized samples were prepared by loading 10 wt% of ILs into dichloromethane. The mixture was added dropwise into dry solid silica. The immobilized silica was dried in oven at 120 °C for 1 h. SAP analysis indicated the pore radius distribution (R_p_) of SBA and SIL are 7.20 and 3.88 nm, respectively. While [BMIM][Cl] and [BMIM][OAc] impregnation showed insignificant effect towards the R_p_ of SIL, the SBA R_p_ showed noticeable reduction. Analysis on XRD showed a similar pattern between pristine and modified samples, which indicates that the silica structure was still preserved after ILs immobilization. The result in the sorption study showed reduction in CO_2_ uptake for all modified silica compared to its pristine material due to reduction in specific surface area and pore volume after immobilization. Among immobilized samples, SBA-OAc and SIL-OAc recorded the highest CO_2_ adsorption performance due to excellent CO_2_ permeation ability provided by [OAc]^−^ anion. The CO_2_/CH_4_ selectivity of the immobilized materials, SBA-Cl and SIL-Cl, improved by 1.51 and 1.37 times, respectively, compared to raw silica. In the CO_2_/CH_4_ selectivity study, the performance of immobilized materials with Cl-based IL were better than acetate-based IL. This is due to the strong interaction existing between the chloride (Cl)^−^ and imidazolium ring which, in turn, provides binding site for CO_2_. To summarize, though modified SBA showed better CO_2_ selectivity, the high cost of commercial SBA should be taken into consideration. Furthermore, the utilization of SIL coupled with [BMIM][Cl] will be the best alternative for development of low-cost adsorbent for CO_2_ capture. 

In the meantime, to overcome the high cost and tedious procedure of task-specific ILs synthesis, Zhang et al. decided to impregnate the mesoporous SBA-15 with PILs, [TEPA][NO_3_] [98]. In their work, the hybrid porous material was prepared by adding 1.0 g of SBA-15 into an ethanol solution containing a designated amount of [TEPA][NO_3_]. The mixture was stirred for 6 h at room temperature. Residue was collected after removing ethanol and further dried under in a vacuum at 100 °C for 12 h. The reduction in the pores’ surface area and diameter of hybrid S15/[TEPA][NO_3_] indicates pore filling by [TEPA][NO_3_]. Crystal phase analysis showed the presence of fraction peaks which correspond to crystallographic planes of SBA-15. As the [TEPA][NO_3_] loading exceeded 50%, the intensity of fraction peak substantially reduced due to a decrease in the 2D hexagonal structure. A large deviation in peak at 0.83° for SBA-15 with 33 and 50% PIL loading is a result of pore swelling due to [TEPA][NO_3_] impregnation. In the study that was conducted at 40 °C and 0.15 bar, the CO_2_ adsorption capacity by [TEPA][NO_3_]/SBA-15 was higher than pristine adsorbent owing to the presence of ILs that provides an additional active site for sorbent interaction. Nevertheless, the breakthrough time and CO_2_ adsorption capacity decreased as IL loading exceeded 66% due to pore blockage and the loss of internal surface area. Regarding the effect of temperature, the CO_2_ adsorption capacity started to decline as the temperature increased above 60 °C due to thermodynamics effect causing CO_2_ desorption rate higher than adsorption capacity. 

On the other hand, Duczinski et al. immobilized two types of IL, [i-C_5_mim] and [BMIM], coupled with several anions, namely, [Cl]^−^, [Tf_2_N]^−^, [PF_6_]^−^ and [DCN]^−^ in commercial mesoporous silica [99]. In this work, the hybrid materials were prepared by dissolving Cl-based ILs in a mixture of toluene and mesoporous silica for 48 h at 95 °C. The unreacted reactant was then removed using Soxhlet extraction, and anion exchange was carried out in the mesoporous silica by using similar procedure as of Karimi et al. and Perdikaki et al. Analysis on solid state ^13^C NMR of modified silica showed the presence of imidazolium ring which indicated the inclusion of ILs. Additional analysis of an NMR relaxation study revealed a more mobile proton population caused by the IL moiety. Besides that, EDS analysis confirmed the successful anion exchange of ILs on grafted mesoporous silica. Furthermore, the surface area and pore volume of modified mesoporous silica was significantly reduced compared to the original material. Immobilizing ILs into mesoporous silica significantly diminishes the CO_2_ uptake capacity due to reduction in surface area and pore volume. However, it has been found that 5 wt% [i-C_5_mim][Tf_2_N] modified silica recorded up to 79.50 ± 70 percent of CO_2_ sorption capacity, virtually equivalent to pristine material (81.70 ± 2.20). The study on the anion effect also demonstrated that ILs with [Tf_2_N]^−^ and [PF_6_]^−^ improve the selectivity CO_2_/N_2_ due to CO_2_ strong affinity towards fluorinated anion.

### 4.4. ILs/MOF

Metal-organic framework (MOF) first came to light on 1995 in an effort to find a new porous solid material that exhibits zeolite-like properties, namely, stability, microporosity, guest exchange and selective catalytic activity [100]. This new class of porous material has a highly-ordered crystalline structure, large surface area and tunable pore size, making it suitable for various applications, such as catalysis, wastewater treatment, and drug delivery [101,102,103]. In the meantime, MOFs can be designed to selectively adsorb CO_2_ by adjusting their pore size and chemical functionality. For example, MIL-53, ZIF-8, HKUST-1, MOF-74 and Uio-66 are some of the commonly used MOFs for CO_2_ capture [104,105,106,107,108]. Magnesium (Mg)-MOF-74 was first synthesized by Caskey and co-workers via solvothermal method and has since become the current benchmark due to its exceptional CO_2_ adsorption capacity, which is 8.8–9.0 mmol/g at ambient condition [10]. The remarkable performance of Mg-MOF-74 is due to its high surface area, 1495 m^2^/g, which is the highest among the MOF-74 group. Additional work that was performed on different metal salts including Zinc (Zn), Nickel (Ni) and Cobalt (Co) towards CO_2_ capture has indicated that Mg-MOF-74 recorded the highest adsorption capacity, 23.6 wt% and 35.2 wt% at 0.1 and 1.0 atm, respectively. However, despite demonstrating remarkable performance, MOFs are susceptible to degradation, particularly after exposure to moisture. Therefore, addressing this weakness has become one of the main challenges, as CO_2_ does contain moisture which, in turn, will negatively impact the MOF performance. 

The incorporation of IL into MOF to produce hybrid porous material is a new approach in producing a new adsorbent with good stability, high CO_2_ affinity and selectivity. In 2011, Chen et al. conducted a computational study on the incorporation of [BMIM][PF_6_] into IRMOF-1 for CO_2_/N_2_ selectivity by using molecular dynamic (MD) and Monte Carlo (MC) simulation [37]. The calculation on radial distribution function (RDF) revealed the structure of [BMIM][PF_6_] is in ordered due to confinement effect of IRMOF-1. Besides that, as the bulky chain of [BMIM]^+^ preferentially resides on the open pore of IRMOF-1, the smaller [PF_6_]^−^ anion had formed a strong interaction with the framework, thus residing in the metal cluster corner. In the meantime, the study on CO_2_ capture by IRMOF-1/[BMIM][PF_6_] was simulated by mimicking flue gas consisting of a 15:85 CO_2_/N_2_ mixture. According to structure analysis, CO_2_ is attracted to the sites of metal cluster of pristine MOF. The presence of [BMIM][PF_6_] resulted in higher CO_2_ adsorption capacity compared to N_2_. This is due to the strong affinity between CO_2_ and [PF_6_]^−^. Meanwhile, increasing the weight ratio of IL incorporating into IRMOF-1 indicates an increase in CO_2_ selectivity that was caused by the additional presence of a favorable site, which is [PF_6_]^−^. Analysis on a CO_2_ isotherm for pristine IRMOF-1 and IRMOF-1/[BMIM][PF_6_] showed that, the hybrid material has a better CO_2_ capacity at low pressure owing to the presence of narrow constrained pores resulting from IL addition.

In other works, Polat and co-workers combined both simulation and experimental works in order to gain a better understanding on the types of force field that possibly influence the CO_2_ capture [109]. The grand conical MC (GCMC) simulation was used to demonstrate two generic force fields, Universal Force Field (UFF) and Dreiding, in order to achieve better prediction performance of nine Cu-BTC/IL materials toward CO_2_, CH_4_ and N_2_ adsorption. A total of seven [BMIM] ILs coupled with various anions, [PF_6_]^−^, trifluoromethanesulfonate [CF_3_SO_3_]^−^, methylsulfate [MeSO_4_]^−^, methanesulfonate [MeSO_3_]^−^, hexafluoroantimonate [SbF_6_]^−^ and octysulfate [OcSO_4_]^−^, were selected to combine with Cu-BTC. The results indicated the gas uptake for simulation conducted by using Dreiding force field was in a good agreement with experimental work for all gases. The study was further explored by synthesis of new combination of Cu-BTC/ILs to confirm the transferability of this approach on other composites. Two [BMIM] ILs with dibutylphosphate [DBP]^−^ and dicyanamide [DCA]^−^ were selected to be combined with Cu-BTC. Before undergoing an adsorption test, both materials were characterized. Analysis on XRD revealed the crystal structure of Cu-BTC was preserved upon the introduction of ILs. In terms of gas separation performance, both materials showed better selectivity in CO_2_/CH_4_, CO_2_/N_2_ and CH_4_/N_2_ compared to pristine MOF low pressure conditions (0.5–10 bar). The result was also in agreement with simulation data obtained by using a Dreiding force field. This shows that the type of computational method that was proposed initially is reliable for gas uptake prediction. However, in terms of surface area and porosity obtained from BET, it had been determined that the CO_2_, CH_4_ and N_2_ adsorption by hybrid materials were lower than pristine Cu-BTC. 

Xia and co-workers synthesized MOF/IL hybrid materials by combining two MOFs, UiO-66 and Nu-1000 with AAILs [41]. The AAILs were [EMIM][Gly] and [EMIM][Phe]. The experimental work was accompanied by simulation in order to obtain better understanding due to the inconsistent results on CO_2_ uptake by both studies. The XRD pattern showed undisrupted MOF framework for all concentrations of IL loading (5, 10 and 30 vol%). Moreover, the addition of ILs into MOF led to a reduction in material surface area and pore volume. Regardless of the amount of AAILs added, the CO_2_ uptake of hybrid material was lower than pristine MOF. This could possibly be due to pores blocking caused by the presence of ILs, thus preventing the entrance of CO_2_ in MOF framework. A study on pore size distribution (PSD) revealed the accumulation of AAILs occurred greatly on one channel of MOF, mainly in pore windows which directly affected the number of active sites for CO_2_ adsorption. The authors also conducted a study by employing GCMC simulation for Nu-1000/[EMIM][Gly] in which two models were adopted, namely, random and blocked. For pristine Nu-1000, both models showed good agreement with experimental work. However, for Nu-1000/[EMIM][Gly], the CO_2_ capture demonstrated by simulation showed opposite outcome with experimental result. While the experimental results indicate the opposite trend, both models suggest that Nu-1000/[EMIM][Gly] exhibits a greater CO_2_ uptake compared to pristine Nu-1000. Additionally, it was identified that blocked models produced closer outcome to experimental result, thus confirming the existence of block pore in MOF. However, this finding could differ depending on ILs and MOF types. 

In 2019, Fereira et al. conducted a study on ILs impregnation onto ZIF-8 for CO_2_/CH_4_ separation [110]. A total of 10 ILs with different alkyl chains and anion were selected to be loaded into ZIF-8. Textural analysis on ZIF-8/ILs materials revealed a reduction in total pore volume and surface areas compared to pristine MOF due to blockage effect caused by IL incorporation. However, the values for total pore volume and surface area are almost similar for all hybrid materials resulting from similar ILs’ molar composition impregnated into ZIF-8. The XRD pattern of ZIF-8/ILs indicates no structural changes occurred when compared to pristine ZIF-8. However, the reduction in the peak intensity at 7.5° and 16.5° for all complexes indicates changes in ZIF-8 electron density. Furthermore, functional group analysis showed changes in peak shifting and intensity that are believed to be caused by the interaction between ILs and ZIF-8. This IL-MOF interaction has also been confirmed by TGA analysis in which ZIF-8/ILs TG curves showed two-step decomposition instead of single-stage mass loss as observed in pristine ZIF-8. Additional analysis on ^19^F NMR revealed ILs with [Tf_2_N]^−^ anion could be found in exterior structure of ZIF-8. Meanwhile, a study on CO_2_ and CH_4_ separation was conducted in terms of individual isotherm and was later used to calculate the ideal selectivity. The adsorption of hybrid ZIF-8/ILs at high pressures was lower than pristine ZIF-8. This is directly related to the reduction in total pore volume and BET surface area. Nevertheless, ZIF-8/[EMIM][OAc] presented an 8.0% increase in adsorption at low pressure (0.5 bar) mainly caused by the [OAc]^−^ chemical interaction with CO_2_. For the ZIF-8 coupled with Tf_2_N-based ILs, the CO_2_ adsorption had reduced as the cation alkyl chain increased. This result is contrary to several previous studies conducted on CO_2_ absorption by ILs alone in which the longer the alkyl chain, the higher the CO_2_ sorbent capacity [26,27]. In terms of MOF/ILs, the flexibility of a long alkyl side-chain has led to additional pore blockage compared to ILs with shorter chain which, in turn, reduces gas accessibility to the available pores. For functional group comparison, the combination of ZIF-8 and hydroxyl ILs produced lower CO_2_ adsorption than ZIF-8/[EMIM][Tf_2_N]. As non-polar CO_2_ and CH_4_ were preferable to be attracted to less polar ILs, the presence of a polar hydroxyl group (OH) in ILs caused the repulsion force with both gases thus lead to poor adsorption capacity. Although the CO_2_ uptake of all hybrid material was less than pristine ZIF-8, the calculated ideal selectivity indicates the selectivity increased up to 40% at 0.5 bar mainly for ZIF-8/[EMIM][OAc] and ZIF-8/[HMIM][DCN].

### 4.5. ILs/Cellulose

Known as the most abundant natural occurring polymer, cellulose building blocks consist of series of glucose chains that are linked by *β*-glycosidic linkages. Cellulose has been seen as a potential alternative sorbent for CO_2_ removal as it exhibits the excellent properties of being low-cost, renewable, non-toxic, biocompatible and biodegradable. Several strategies including cellulose chemical modification and incorporation of inorganic nanoparticle into nanocellulose were proposed to enhance their selectivity towards CO_2_. Bernard and co-workers suggested a new approach on utilizing cellulose extracted from rice husk as a solid support for CO_2_ capture in an effort to overcome the drawbacks posed by amines. It was discovered that the combination of extracted cellulose with JEFFAMINE^®^ D-400 Polyetheramine showed 1.091 and 0.409 mmol CO_2_/g of CO_2_ uptake at 10 and 1 bar, respectively [111]. In another study, Miao and co-workers employed cellulose aerogels constructed from old corrugated containers for CO_2_ capture at ambient conditions [112]. This work indicates that the CO_2_ adsorption capacity of cellulose aerogels was in the range of 1.96–11.78 mmol/g. Though cellulose showed great potential as a renewable adsorbent for CO_2_ removal, there are several drawbacks that need to be considered. Up to this time, the structural strength and thermal stability of cellulose are still not meeting the requirement in CO_2_ adsorption [113]. Meanwhile, the process for modifying cellulose pores involving various processes, such as silanization [114] and esterification [115], has a relatively high cost.

Bernard and co-workers explored the use of cationic cellulosic poly(ILs) to improve CO_2_ sorption [116]. For this study, imidazolium and ammonium cations coupled with different anions such as [Cl]^−^, [Tf_2_N]^−^, [BF_4_]^−^ and [PF_6_]^−^ were selected to be combined with extracted cellulose. Prior to synthesis of cellulose-based poly(ILs), cellulose was extracted from rice husk and underwent modification using SOCl_2_ to produce chlorinated cellulose (CDC). The cellulose-based poly(ILs) was prepared by adding CDC into 1-methylimidazole or triethylamine to produce [Celmim][Cl] and [CelEt_3_N][Cl] precipitates. The ILs precipitate was purified with methanol before drying under vacuum at 60 °C. In the meantime, anion exchange was conducted at room temperature in designated solvent to produce [Celmim][Tf_2_N], [Celmim][BF_4_], [Celmim][PF_6_], [CelEt_3_N][Tf_2_N], [CelEt_3_N][BF_4_] and [CelEt_3_N][PF_6_]. Analysis on ^13^C NMR confirmed the structures of cellulosic poly(ILs). On the other hand, XPS data revealed the presence of the atoms from all anions with traces of impurities came from SiO_2_ of rice husk, and not all halogenated carbons were likely subjected to the reaction with cation. XRD pattern revealed drastic reduction in crystallinity of cellulosic poly(ILs) compared to cellulose. The result of CO_2_ uptake capacity showed the cellulose functionalization promotes CO_2_ affinity towards the adsorbent. For example, the CO_2_ uptake capacity of [CelEt_3_N][PF_6_] and cellulose at 3.0 MPa and 0 °C were 88 and 168 mg/g, respectively. Moreover, it has been found that [CelEt_3_N][PF_6_] with [PF_6_]^−^ anion displayed superior CO_2_ uptake compared to other anion attributed by well distributed of fluorine atom in cellulose space. The evaluation on [CelEt_3_N][PF_6_] reusability at 0.1 MPa demonstrated the constant sorption value in all five cycles indicates the robustness of the material for CO_2_ removal. 

In other work, Reed and co-workers investigated the pressure swing CO_2_ capture using cellulose-supported ILs [117]. Their study mainly focuses on combination of cellulose with 5–50 wt% solid [N_444_][Oac] due to its easy handling, high water tolerance and effectively coating cellulose. In this work, the hybrid sorbent was prepared by dissolving ILs in solution containing equal ratio of methanol and isopropanol. Then, cellulose was added into the mixture and solvent was removed under vacuum resulting in coated cellulose. As the capacity increased linearly with rising pressure, a noticeable enhancement in CO_2_ uptake capacity was observed at a pressure of 10 bar for the 25:75 weight ratio of [N_444_][Oac]: cellulose. At 30 bar, the CO_2_ uptake capacity reached up to 3.16 wt% which is about 8.5 times higher than pure ILs. Morphological analysis revealed the presence of surface roughness which was induced by the presence of [N_444_][Oac] in hybrid sorbent, thus creating a pseudo-microporous structure that can provide CO_2_ binding sites. This assists in improving CO_2_ capture capacity as observed in [N_444_][Oac]/cellulose. However, the uptake capacity was gradually decreased starting from 80 to 95 wt% of cellulose loading which could possibly be due to incomplete [N_444_][Oac] coverage on cellulose surface, thus leading to a smaller CO_2_ capture area. The study on CO_2_ capture was extended to [N_1888_][Oac]/cellulose, [N_888_][Br]/cellulose and [Bmim][Br]/cellulose. For example, pure [N_888_][Br] had the lowest CO_2_ uptake of 0.71 wt%, whereas [N_888_][Br]: cellulose (30:70) exhibited a significant improvement with a CO_2_ capacity of 1.98 wt%. The coating of ILs onto cellulose resulted in an improvement of CO_2_ capture compared to pure ILs. Despite the fact that [N_1888_][Oac]/cellulose exhibited an enhancement in surface area, the crucial factor in CO_2_ capture is believed to be the surface properties, given the slight increase observed in comparison to pure [N_1888_][Oac]. In the meantime, additional study on adsorption and desorption showed the sorbent is highly stable while simultaneously maintaining the CO_2_ uptake capacity. 

Another study tailored the physiochemical properties of chitosan-aerogel with several poly-ILs (PILs), namely, P[DADMA][Cl], P[DADMA][OAc], P[VBA][Cl] and P[VBMPyr][Cl] [118]. This combination produces a material known as AEROPIL which will be used for CO_2_ capture. In their work, two types of AEROPIL, with and without glutaraldehyde, were prepared by the sol–gel method. Chitosan was initially dissolved in water containing 1.0% (*v*/*v*) acetic acid. A solution of PILs was incorporated into the gel solution subjected to the concentration of chitosan and glutaraldehyde. The mixture was transferred into a plastic syringe and slowly dropped in gelation bath (1.0 mol/L NaOH) solution at flow rate 0.65 mL/min. The hydrogel beads were allowed to remain in the gelation bath for 24 h, after which the solution was replaced with ethanol to remove any remaining water traces. The alcogel particles were then collected and placed in an autoclave in which the solvent was removed by using supercritical extraction method under controlled pressure and temperature. FTIR and ^13^C solid NMR confirmed the successful of PILs incorporation into chitosan aerogel. The analysis of the textural characteristics showed that AEROPIL-15%P[DADMA][OAc] and AEROPIL-15%P[DADMA][Cl] have higher specific surface area and pore volume than the pure chitosan aerogel (CHT). This is probably due to hydrophilic properties of P[DADMA] ILs which allow better affinity towards chitosan. Conversely, the AEROPILs that were modified with vinylbenzyl PILs experienced the opposite effect. The CO_2_ capture was conducted by using thermogravimetric analyzer (TGA) in which the test was performed under ambient conditions. The incorporation of 30% P[DADMA][Cl] into AEROPIL resulted in a CO_2_ capacity of 0.70 mmol/g after just 10 min of exposure time. According to the authors, this is 35 times greater than the CO_2_ capture capacity of pure chitosan with the value of 0.02 mmol/g.

## 5. Challenges in IL-Hybrid Materials

The hybridization of ILs has enabled the customization of the properties of pristine material. One key advantage of ILs hybridization is the improvement in CO_2_ selectivity of the material during gas capture process. Apart from enhancing the thermal stability of the material, the incorporation of ILs has effectively reduced the heat capacity of the sorbent. However, the benefits of ILs hybridization are accompanied by challenges that are crucial and have to be addressed. 

Compatibility has become a hurdle in hybridization as ILs and other materials may pose differences in chemical structure, solubility behavior and physical properties. Therefore, the search for a suitable combination which will not compromise the original structure and composition of base material has become an utmost important aspect in ILs hybridization. For example, in the case of ILs/MOF hybrid material, the hydrophobicity or hydrophilicity nature of ILs plays a crucial role. Hydrophilic ILs can lead to the collapse of the MOF structure due to their sensitivity to moisture. Therefore, selecting hydrophobic ILs has become essential to prevent any adverse effects on the stability of the MOF structure. Other than that, the separation and regeneration process of captured CO_2_ from hybrid materials can be challenging due to the presence of various interactions such as intermolecular forces, ionic interactions and covalent bonds. In addition, scaling up the ILs hybridization process to the industrial level could be intricate as various factors such as ILs availability, material purity, durability of materials and process efficiency must be taken into consideration to ensure economic feasibility in gas capture process. 

## 6. Conclusions

Research related to ILs utilization for CO_2_ capture has been ongoing for more than two decades. Based on preceding discussions, the research landscape surrounding utilization of ILs as sorbents for CO_2_ capture is continuously advancing with a wide array of different classes of ILs being synthesized and studied. In general, ILs have undergone various structural modifications in which each class was developed to address the limitations of the earlier generation of ILs. To further enhance ILs performance, a new method known as ILs hybridization was proposed with the ultimate goal of developing sustainable solutions for CO_2_ capture. Absorbent mixtures containing ILs/amine blending exhibit lower viscosity and better thermal stability compared to the pristine materials. Besides that, the addition of amine into IL has significantly improved the CO_2_ absorption capacity. The incorporation of ILs into amines also reduces the energy requirements during the CO_2_ desorption and absorbent regeneration process. Meanwhile, the incorporation of ILs into porous materials such as activated carbon, mesoporous silica and MOF has led to pore blocking, thus resulting in a reduction in the adsorbent’s surface area. As the gas adsorption performance is highly dependent on the total surface area, the presence of ILs within the pores of porous materials leads to a significant reduction in CO_2_ adsorption capacity. However, the selectivity of adsorbent towards CO_2_ increases compared to pristine material. In the case of ILs/cellulose hybrid material, the morphology of cellulose has been altered after the introduction of ILs. While the IL/cellulose combination exhibits good CO_2_ solubility, ILs anions do still play a significant role in determining the overall adsorption capacity. In the meantime, the utilization of cellulose as solid support has also significantly reduced the ILs amount usage in CO_2_ capture. 

On top of that, among the various ILs hybrid materials discussed in the previous section, IL/cellulose emerges as a particularly promising candidate for industrial-scale applications. Its potential is underscored not only by its promising performance towards CO_2_ capture, but also by the abundant availability of lignocellulosic biomass which serves as the primary source of cellulose. This approach not only mitigates environmental concerns linked to biomass waste disposal, but also presents a sustainable and viable solution. Additionally, the blending of amine/ILs also holds significant promise for the industry. The technology involving the use of amines as CO_2_ absorbents has been well-developed and extensively studied. Incorporating ILs into amine solutions offers a cost-effective solution, potentially eliminating the need for extensive modifications to existing CO_2_ capture infrastructure to accommodate the blended mixture.

ILs hybridization offers a promising approach to overcome ILs’ main limitation, which is viscosity. Through various processes of ILs hybridization, such as blending, encapsulation and immobilization, novel ILs hybrid material with better stability, selectivity and functionality for CO_2_ capture has been produced. Additionally, hybridization not only enhances the properties of the individual ILs, but also contributes to the improvement of the pristine material’s properties. However, several recommendations have been suggested to improve the research for its potential application at industrial scale. Current study focuses on lab-scale production of hybrid materials. Therefore, comprehensive study on developing IL hybrid material that are compatible with the existing system of CO_2_ capture is required. Concurrently, it is recommended to conduct a study related to the environmental implications of employing ILs hybrid materials, with particular emphasis on the characterization of degradation products and the development of effective waste management strategies. Last but not least, conducting Life Cycle Assessment (LCA) analysis on ILs hybrid material for CO_2_ capture can provide a valuable insight into the process optimization and overall sustainability of CO_2_ removal strategies. 

## Figures and Tables

**Table 1:** CO_2_ sorption by various ILs classes.

		Conditions			CO_2_ Absorption Capacity			
Class of ILs	ILs	*T* (°C)	*P* (bar)	*t* (min)	Mole Fraction	molCO_2_/mol IL	wt%	Ref.
Conventional RTILs	[BMIM][PF_6_]	^___e^	80	^___e^	0.600	^___e^	^___e^	[18]
	[BMIM][PF_6_]	25	13	^___e^	~0.210	^___e^	^___e^	[23]
	[BMIM][PF_6_]	40	93	^___e^	0.720	^___e^	^___e^	[22]
	[BMMIM][PF_6_]	25	13	^___e^	~0.190	^___e^	^___e^	[23]
	[EMIM][Tf_2_N]	25	13	^___e^	~0.280	^___e^	^___e^	[23]
	[EMMIM][Tf_2_N]	25	13	^___e^	~0.260	^___e^	^___e^	[23]
	[HMIM][Tf_2_N]	40	83.7	^___e^	0.720	^___e^	^___e^	[27]
	[OMIM][Tf_2_N]	40	83.7	^___e^	0.763	^___e^	^___e^	[27]
TSILs	[AEmim][Tf_2_N]	30	1.6	^___e^	^___e^	0.490	^___e^	[28]
	[AEmim][BF_4_]	30	1.6	^___e^	^___e^	0.425	^___e^	[28]
	[AEmim][DCA]	30	1.6	^___e^	^___e^	0.435	^___e^	[28]
	[P_2228_][6BrInda]	58.9	0.83	^___e^	^___e^	~1.00	^___e^	[31]
	[DMAPAH][4F-PhO]	30	1.5	^___e^	^___e^	0.86	^___e^	[32]
	[E_1_Py][C(CN)_3_]	30	20	^___e^	0.473	^___e^	^___e^	[29]
	[E_1_Py][N(CN)_2_]	30	20	^___e^	0.382	^___e^	^___e^	[29]
	[E_1_Py][SCN]	30	20	^___e^	0.184	^___e^	^___e^	[29]
	aq-[APmim][Br]	30	^___e^	^___e^	^___e^	0.65	^___e^	[43]
Polymer-ILs	[MDAP][TF_2_N]-PDC	0	1.0	^___e^	^___e^	^___e^	1.390	[34]
	[PBIMT][BF_4_]	22	0.80	600	^___e^	^___e^	0.241	[33]
	[PVBIM][PF_6_]	22	0.80	600	^___e^	^___e^	0.322	[33]
	[PVBIM][BF_4_]	22	0.80	600	^___e^	^___e^	0.305	[33]
	HPIL-Cl (1)	25	1.0	^___e^	^___e^	^___e^	5.30	[35]
AAILs	[P_66614_][Pro]	25	1.01	^___e^	^___e^	0.91	^___e^	[40]
	[P_66614_][Met]	25	1.01	^___e^	^___e^	0.99	^___e^	[40]
	[P_66614_][Gly]	25	1.01	^___e^	^___e^	0.97	^___e^	[40]
	[B_4_Pyr][Arg]	25	6.0	^___e^	0.613	^___e^	^___e^	[38]
	[B_4_Pyr][Gly]	25	6.0	^___e^	0.475	^___e^	^___e^	[38]
	[N_66614_][Lys]	25	1.0	1440	^___e^	2.1	13.1	[50]
	[N_66614_][Lys]	80	1.0	1440	^___e^	1.0	^___e^	[50]
Dual-functionalized ILs	aq-[APmim][Gly]	30	^___e^	^___e^	^___e^	1.23	^___e^	[43]
	[N_1,1,6,2O4_][Lys]	20	1.0	200	^___e^	1.62	19.02	[46]
PILs	[MTBDH][HFPD_2_]	23	1.0	60	^___e^	2.04	^___e^	[48]
	[TMGH][Pyrr]	40	1.0	~80	^___e^	0.66	^___e^	[49]
	[TMGH][Im]	40	1.0	~80	^___e^	0.64	^___e^	[49]

^__e^ the data were not mentioned in literature.

**Table 2:** Advantages and disadvantages of IL hybridization method.

Methods	Advantages	Disadvantages	Examples of IL-Hybrid Materials	Ref.
Blending	Straightforward and simple method	Restricted to material that existed in the same phase (liquid) Mixing material with different chemical properties could lead to non-homogenized mixture or precipitate formation	EDA/[DEAPOAc], ChoCl: 1, 4-butanediol (1: 5)/[BMIMOTF]	[73,74]
Immobilization	Short processing time thus allowing rapid production of material Minimal amount of equipment utilization	Additional purification step which can increase the production cost Achieving consistent immobilization efficiency by maintaining batch-to-batch reproducibility, can be technically demanding and require additional optimization study.	SiO_2_/[ASBI][TfO], SiO_2_/[ASBI][HSO_4_] and SBA-15/multifunctional Ils,	[75,76]
Wet impregnation	Versatility in materials combination Practical for industrial application due to straightforward preparation process Low cost Limited amount of waste	Loss of material during impregnation process The resulted ILs loading into solid support may fall below the target value. Organic solvent is required as reaction medium	MIL-101/[EMIM][Oac], MOF-177/[EMIM][Oac]	[77,78]
Polymerization	Improved the stability and durability of material	Present challenges in identifying suitable ILs to act as monomers or for combination with existing monomers to form polymer materials Complicated synthesis route	polyILs@MIL-101	[71]
Ionothermal synthesis	Access to new material with distinct properties than pristine material Minimize the utilization of organic solvent as ILs already act as reaction medium	Limited selection in material combination Complex reaction mechanism as different IL could resulted in different material topology Slow reaction rates due to high viscosity of ILs Pose challenges in product recovery	Cu_3_(tpt)_4_(BF_4_)_3_.(tpt)_2/3_.5H_2_O [tpt = 2,4,6-tris(4-pyridyl)-1,3,5-triazine], MOF-5 (IL), [Co_2_Na(bptc)_2_][EMIm]_3_	[79,80]

## Data Availability

Not applicable.

## Sample Availability

Not available.

## Abbreviations

MDEA	Methyldiethanolamine
DEA	Diethanolamine
MEA	Monoethanolamine
EDA	Ethanoldiamine
DETA	Dietylenetriamine
DBU	1,8-Diazabicyclo[5.4.0]undec-7-ene
DABCO	1,4-diazabicyclo[2.2.2]octane
[Cho][Gly]	Choline glycinate
[BMIM][PF_6_]	1-butyl-3-methylimidazolium hexafluorophosphate
[EMMIM][Tf_2_N]	2,3-dimethyl-1-ethylimidazolium bistrifluoromethyl(sulfonyl)imide
[EMIM][Tf_2_N]	1-ethyl-3-methylimidazolium bistrifluoromethyl(sulfonyl)imide
[2-AEmim][Tf_2_N]	1-(3-aminoethyl)-3-methylimidazolium bistrifluoromethyl(sulfonyl)imide
[PVBIM][BF_4_]	Poly 1-(4-vinylbenzyl)-3-butylimidazolium tetrafluoroborate
[BMIM][BF_4_]	1-butyl-3-methylimidazolium tetrafluoroborate
[PVBIM][PF_6_]	Poly 1-(4-vinylbenzyl)-3-butylimidazolium hexafluorophosphate
[PBIMT][BF_4_]	Poly 2-(1-butylimidazolium-3-yl)ethyl methacrylate tetrafluoroborate
[MDAP][Tf_2_N]-PDC	Methyl-diaminopyridinium bis(trifluoromethanesulfonyl)imide-(2,6-pyridinedicarbonyl chloride)
[P(C_4_)_4_][Gly]	Tetrabutylphosphonium glycinate
[P_66614_][Pro]	Trihexyl(tetradecyl)phosphonium prolinate
[P_4444_][Pro]	Tetrabutylphoshonium prolinate
[B_4_MPyr][Arg]	1-butyl-4-methyl pyridinium arginate
[C_1_ImPA][Gly]	1-(2-aminopropyl)-3-methylimidazolium glycinate
[TEPA][Im]	Tetraehylnepentamine imidazolate
[TETAH][Pz]	Triethylenetetramine 1,2,4-triazolate
[TMGH][Pyrr]	Tetramethylgunidinium pyrrole
[TMGH][Im]	Tetramethylgunidinium imidazole
[TBP][MeSO_3_]	Tetrabutylphsphonium methanesulfonate
[TETAH][Ly]	triethylenetetramine L-lysine
[VEIM][Br]	1-vinyl-3-ethylimidazolium bromide
[EMIM][Br]	1-ethyl-3-methylimidazolium bromide
[BMIM][OTF]	1-butyl-3-methylimidazolium trifluoromethanesulfonate
[ASBI[TfO]	3-allyl-1- (4-sulphobutyl)imidazolium triflate
[ASBI][HSO_4_]	3-allyl-1- (4-sulphobutyl)imidazolium sulphate
[EMIM][OAc]	1-ethyl-3-methylimidazolium acetate
[BEIM][BF_4_]	1-butyl-2-ethylimidazolium tetrafluoroborate
[DMAPAH][OAc]	3-dimethylamino-1-propylamine acetate
[BMIM][OAc]	1-butyl-3-methylimidazolium acetate
[EMIM][OcSO_4_]	1-butyl-3-methylimidazolium octylsulphate
[HMIM][BF_4_]	1-hexyl-3-methylimidazolium tetrafluoroborate
[EMIM][Gly]	1-ethyl-3-methylimidazolium glycinate
[P_8883_][Tf_2_N]	Phosphonium bis-trifluoromethanesulfonimide
[VBTMA][Arg]	4-(vinylbenzyltrimethyl)ammonium argininate
[VBTMA][Lys]	4-(vinylbenzyltrimethyl)ammonium lysinate
[VBTMA][Gly]	4-(vinylbenzyltrimethyl)ammonium glycinate
[BMIM][Cl]	1-butyl-3-methylimidazolium chloride
[TEPA][NO_3_]	Tetraethylenepentammonium nitrate
[i-C_5_mim][Tf_2_N]	1-isopentyl-3-methylimidazolium bis-trifluoromethanesulfonimide
[EMIM][Phe]	1-ethyl-3-methylimidazolium phenolate
[HMIM][DCN]	1-hexyl-3-methylimidazolium dicynamide
[Celmim][Cl]	Cellulosic-6-imidazolium chloride
[CelEt_3_N][Cl]	Cellulosic-6-triethylammonium chloride
[Celmim][Tf_2_N]	Cellulosic-6-imidazolium bis-trifluoromethanesulfonimide
[Celmim][BF_4_]	Cellulosic-6-imidazolium tetrafluoroborate
[Celmim][PF_6_]	Cellulosic-6-imidazolium hexafluorophosphate
[CelEt_3_N][BF_4_]	Cellulosic-6-triethylammonium tetrafluoroborate
[CelEt_3_N][PF_6_]	Cellulosic-6-triethylammonium hexafluorophosphate
[N_444_][Oac]	Tetrabutylammonium acetate
[N_1888_][Oac]	Tetraoctylmethylammonium acetate
[N_888_][Br]	Tetraoctylammonium bromide
P[DADMA][Cl]	Poly(diallyldimethylammonium) acetate
P[DADMA][OAc]	Poly(diallyldimethylammonium) acetate
P[VBA][Cl]	Poly(*p*-vinylbenzyltriethylammonium) chloride
P[VBMPyr][Cl]	Poly(1-methyl-1-(4′-vinylbenzyl)pyrrolidinium) chloride
[P_2228_][6BrInda]	triethyl(octyl)phosphonium 6-bromoindazolide
[DMAPAH][4F-PhO]	N,N-dimethyl-1,3-propane diamine 3-fluorophenolate
